# Development of an Individualized Immune Prognostic Signature for Clear Cell Renal Cell Carcinoma through the Identification of Differential Immune Genes

**DOI:** 10.1155/2021/9587084

**Published:** 2021-08-11

**Authors:** Jianfeng Wang, Chaozhi Tang, Xiaowu Liu

**Affiliations:** Department of Urology, The First Affiliated Hospital of China Medical University, Shenyang 110001, China

## Abstract

Increasing evidence has shown that tumor microenvironments are an important feature in clear cell renal cell carcinoma (ccRCC) carcinogenesis and therapeutic efficacy. In this study, two subtypes of ccRCC, high- and low-immune groups, were identified based on the immune gene datasets, of which the differential immune genes were identified accordingly. Furthermore, we constructed a risk prognosis model using five immune genes, specifically, AQP9, KIAA1429, HAMP, CCL13, and CCL21. This model was highly predictive of ccRCC clinical characteristics and showed potential for use in immunotherapy. Furthermore, the five identified genes were highly correlated with the abundance of B cells, CD4 T cells, CD8 T cells, macrophages, neutrophils, and dendritic cells in the tumor microenvironments. Among them, AQP9, KIAA1429, and HAMP exhibited significant prognostic potential. These findings indicate that monitoring and operating tumor microenvironments are of great significance for ccRCC prognosis and precise immunotherapy.

## 1. Introduction

Renal cell carcinoma (RCC) accounts for more than 330 000 cases of cancer worldwide and 140 000 cancer-related deaths each year. The incidence of kidney cancer has been steadily increasing over the past few decades. In the United States alone, more than 14 000 kidney cancer-related deaths occur each year [1]. While all types of RCC are nephron-derived and receive similar clinical treatments, the various histological subtypes are highly heterogeneous in terms of biology and susceptibility to therapy. Among these subtypes, clear cell RCC (ccRCC) is the most common (70–80%) and is one of the most aggressive subtypes [2].

The tumor microenvironment is essential for the initiation and maintenance of tumorigenesis [3]. It is composed of tumor cells, extracellular matrix (ECM), signal molecules, stromal cells (such as fibroblasts, vascular endothelial cells, and pericytes), and immune cells [4, 5]. Numerous studies have indicated that the tumor microenvironment is directly involved in the development of drug resistance to targeted therapies. Tumor-infiltrating cells can have either tumor-suppressing or tumor-promoting effects depending on the tumor type or model. For example, regulatory T cells (Treg cells), bone marrow-derived suppressor cells, and tumor-associated macrophages are associated with protumor functions [6, 7]. Recently, there has been evidence showing that T cell activation status is a key determinant of the prognosis of ccRCC and may be a key factor in the response to immunotherapy in highly invasive ccRCC tumors. Furthermore, the levels of CD8+ T cells have been found to be associated with improved clinical results and related to immunotherapy response [8, 9].

The lack of sensitivity of ccRCC to chemotherapy and radiation therapy has prompted research into new treatment options. Clinical knowledge has shown that ccRCC is a tumor rich in blood vessels. Thus, targeting the von Hippel–Lindau (VHL) protein, which promotes angiogenesis mainly by regulating vascular endothelial growth factor, is an attractive method for the treatment of sporadic ccRCC [10]. Furthermore, major breakthroughs in ccRCC treatment have been achieved with the development of targeted therapeutic agents, including multitarget tyrosine kinase inhibitors and mTOR inhibitors. Recently, other treatment strategies, including immune checkpoint inhibitors, have also been found to be effective treatment options for advanced ccRCC. In addition, the latest advancements in research on disease biology, tumor microenvironments, and drug resistance mechanisms have laid the foundation for attempts to combine targeted therapy with a new generation of immunotherapy, taking advantage of a possible synergy.

In this study, we systematically explored the differences in immune cells or signature infiltrations in ccRCC data. We also studied the complex biological functions, immune processes involved, and networks that regulate these molecules. In addition, we systematically studied epigenetic characteristics related to the risk of developing ccRCC, such as chemosensitivity, immunotherapy sensitivity, mutation, and methylation. This study aids in the promotion of precise and personalized treatment strategies for patients with ccRCC, providing clinical guidance.

## 2. Materials and Methods

### 2.1. Data Processing

Kidney renal clear cell carcinoma (KIRC) datasets were obtained from The Cancer Genome Atlas (TCGA) and ArrayExpress databases. RNA-seq data (fragments per kilobase of transcript per million mapped reads (FPKM)) from the 72 normal samples and 539 cancer samples of the TCGA database, variant data of VarScan, methylation data, and clinical information were downloaded from UCSC Xena (https://xenabrowser.net/datapages/). Gene expression values of samples that were taken from the same patients were averaged. Finally, 530 patients were included in the survival analysis. Two other datasets, E-MTAB-1980 (101 cancer samples) and E-MTAB-3267 (53 cancer samples), were downloaded from the ArrayExpress (https://www.ebi.ac.uk/arrayexpress/) database. The Ensemble database (http://asia.ensembl.org/index.html) was used for gene annotation.

### 2.2. KIRC Subtype, ESTIMATE, and CIBERSORT Analysis

A total of 29 immune gene sets, including different immune cell types, functions, and pathways, were used to represent tumor immunity. Single sample gene set enrichment analysis (ssGSEA) algorithms were performed on the KIRC samples by the GSVA package to evaluate the immunological traits using the R software. The high- and low-immune subtypes of KIRC were identified using the ConsensusClusterPlus package (k-means clustering was used, with 50 iterations, each using 80% of the samples).

The ESTIMATE algorithm [11], using transcriptome data, was used to infer tumor cell composition (TumorPurity), infiltration status of stromal cells (stromal score), and immune cell scores (ImmuneScore) of our samples.

CIBERSORT [12] is a deconvolution algorithm that is based on the principle of linear support vector regression. It was used in our study to calculate the abundance of 22 tumor-infiltrating immune cells in each sample of the expression matrix by using the corresponding immune gene sets.

### 2.3. Differential Immune Gene Selection Based on the High- and Low-Immune Groups

We used the limma package to identify 1012 differential genes in the identified KIRC high-immunity group in the TCGA cohort, with the identified KIRC low-immunity group as a control. The standard used for the differential gene screening was |log2 FC| >1, and a *P* value <0.05 was considered significant. These genes were then intersected with the identified immune genes from the IMMPORT database (https://www.immport.org/) and the genes in the dataset E-MTAB-1980 to obtain a total of 132 differential immunity genes.

### 2.4. Immunity Gene-Associated Prognostic Model

To screen for immune differential genes that significantly affect the survival of KIRC patients, we performed a single-factor Cox regression analysis, using a *P* value <0.05 to indicate statistical significance. From this, a total of 67 immunological differential genes were obtained. To further narrow the gene range for constructing the risk model, we used lasso regression to reduce it to 8 immune differential genes. Finally, we performed multifactor Cox regression analysis to further narrow it down to 5 immune differential genes in the model using *P* value <0.05 as the screening threshold.

### 2.5. Statistical Analysis

Fisher's exact test was used for the Tumor Immune Dysfunction and Exclusion (TIDE) immunotherapy response in the high- and low-risk groups of the TCGA cohort, sunitinib response in the high- and low-risk groups of the E-MTAB-3267 cohort, and the distribution of the clinical characteristics of the high-and low-risk combination. Kaplan–Meier survival analysis was performed using the log rank-sum test. The Kruskal–Wallis test was used for comparing three groups, and the Mann–Whitney *U* test was used for comparing two groups. All tests were two-sided, and the visualization of the data was achieved using R (3.5.3) and GraphPad Prism 8.0.

## 3. Results

### 3.1. Exploration of KIRC Immunophenotypes and Tumor Microenvironments

**Figure 1:**
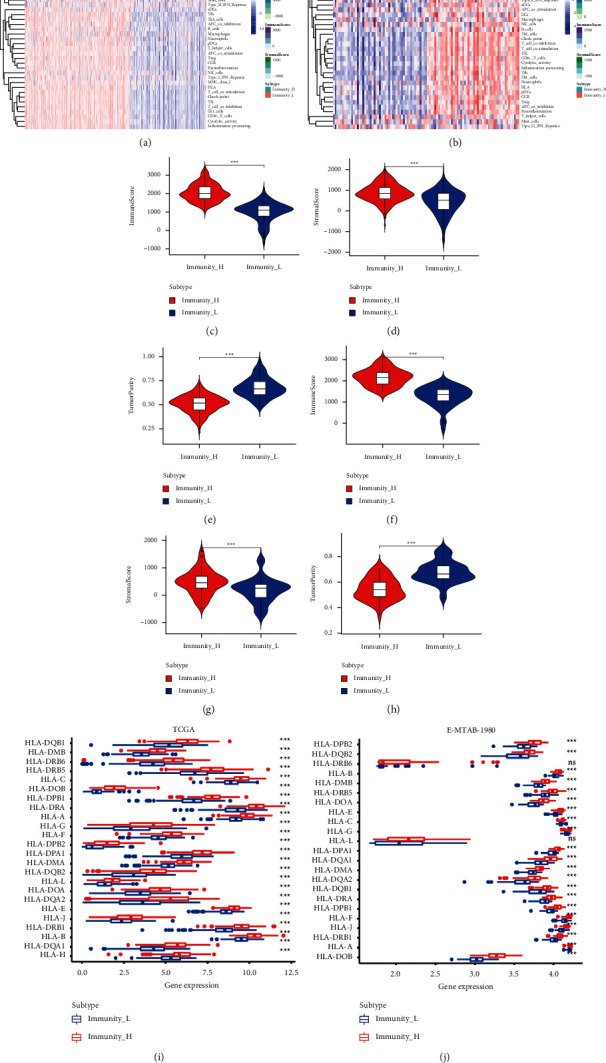
Identification of the immunophenotypes and tumor microenvironments of samples from patients with clear cell renal cell carcinoma (ccRCC). Heatmap of the immune characteristics and tumor microenvironment in the (a) TCGA and (b) E-MTAB-1980 cohorts determined by the ssGSEA algorithm. Consensus clustering analysis, distribution of immune score, stromal score, and tumor purity of the low- and high-immunity groups in the (c–e) TCGA and (f–h) E-MTAB-1980 cohorts. Differences in immunogenicity between high-immunity and low-immunity groups in (i) TCGA and (j) E-MTAB-1980 cohorts. ^*∗∗∗*^*P* < 0.001; ns: no significance.

Based on datasets, each with more than 100 cancer samples, we used 29 immune gene sets that could represent multiple immune cells, functions, and pathways, using the ssGSEA algorithm to score each sample. Consistent clustering was used to identify tumor samples from the TCGA and E-MTAB-1980 databases. According to the consistent cumulative distribution function and Delta Area Plot, results showed that the optimal value was obtained when *K* = 2. Through a heat map analysis, we found that the KIRC samples could be classified into two groups, high- and low-immunity groups. We performed further analysis using ESTIMATE to compare both groups. We found that the high-immunity group had a higher stromal and immune score but had lower tumor purity (Figure 1). Next, we measured the difference in human leukocyte antigen marker levels between the high- and low-immunity groups. We found that the high-immunity group had higher immunogenicity than the low-immunity group.

**Figure 2:**
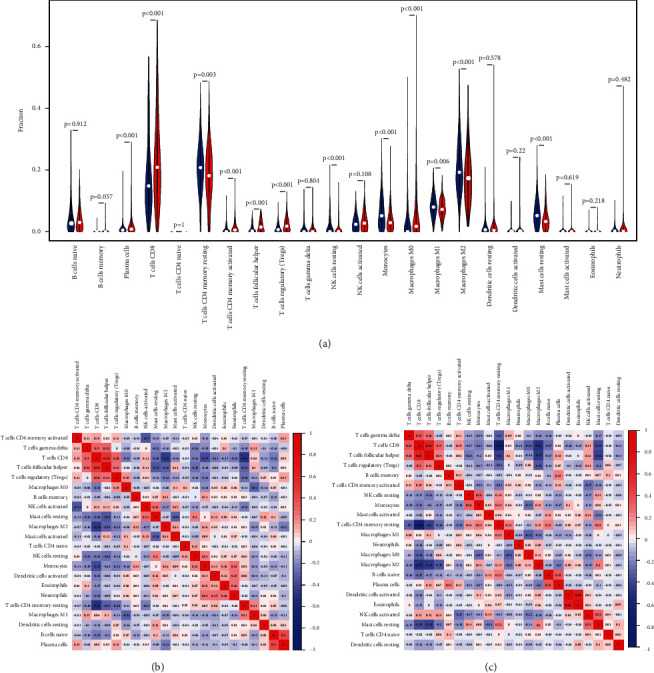
Differences in the tumor microenvironments between the high- and low-immunity groups. (a) Differences in the 22 tumor-infiltrating immune cells between the high- and low-immunity groups. Blue represents the low-immunity group, and red represents the high-immunity group. Correlations between the immune cells in the (b) low-immunity and (c) high-immunity groups.

To identify the specific differences in the tumor microenvironments between the high- and low-immunity groups, we used the CIBERSORT algorithm to measure the differences between the 22 human immune cells of the two groups. The high-immunity group showed significantly higher levels of CD8 T cells, plasma cells, CD4 T cells, T follicular helper cells, regulatory T cells (Tregs), and M0 macrophages, while the low-immunity group had higher levels of monocytes, M1 macrophages, M2 macrophages, and resting mast cells (Figure 2).

### 3.2. Identification of Differential Immune Genes Based on the High- and Low-Immunity Groups

After identifying the heterogeneity of ccRCC tumors, we performed differential analyses on the high- and low-immunity groups to further explore this difference. A total of 360 downregulation and 652 upregulation differences were identified. These genes were then intersected with the identified immune genes from the IMMPORT database (https://www.immport.org/) and the genes in the E-MTAB-1980 dataset, resulting in a total of 132 differential immune genes.

To screen for immune genes that affect patients with KIRC, a single-factor Cox analysis was performed, yielding 67 prognostic immune genes. Functional enrichment analysis of gene ontology (GO) and the Kyoto Encyclopedia of Genes and Genomes (KEGG) revealed that these 67 genes are involved in T cell activation, leukocyte cell-cell adhesion, cytokine activity, chemokine activity, Th1, Th2, and Th17 cell differentiation, the NF-kappa B signaling pathway, the T cell receptor signaling pathway, the JAK-STAT signaling pathway, PD-L1 expression, and the PD-1 checkpoint pathway in cancer (Supplementary Figure 2).

### 3.3. Construction and Validation of an Immunity-Based Risk Signature

To further screen the above genes, lasso regression was used to prevent overfitting between the genes. Finally, a risk regression model based on the immunity-related genes was constructed using multifactor Cox analysis, with the results shown in Table 1. The risk score of each KIRC patient was based on the following formula:

(1) $$\begin{array}{c} \text{risk score}=0.11893\times \text{CCL}13\text{expression}+0.01586\times \text{AQP}9\text{expression}+0.00636\times \\ \text{CCL}21\text{expression}+0.26158\times \text{HAMP}-0.17781\times \text{KIAA}1429\text{expression}. \end{array}$$

Each dataset was based on the risk score of the sample, and the median values were used as the basis for division into the high-and low-risk groups.

**Figure 3:**
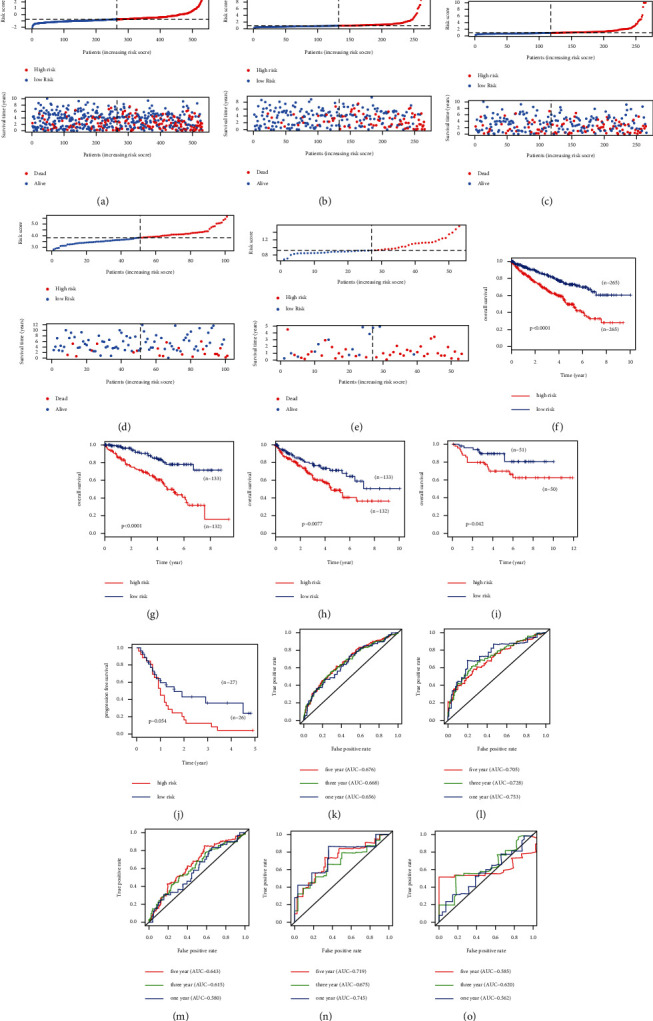
Construction and validation of an immunity-based risk signature. Distribution of the risk score, survival time, and survival status of the patients with KIRC in the (a) TCGA, (b) TCGA-Train, (c) TCGA-Test, (d) E-MTAB-1980, and (e) E-MTAB-3267 cohorts. Kaplan–Meier survival curves based on the risk model in the (f) TCGA, (g) TCGA-Train, (h) TCGA-Test, (i) E-MTAB-1980, and (j) E-MTAB-3267 cohorts. Receiver operating characteristic curve and the corresponding AUCs of the risk model in the (k) TCGA, (l) TCGA-Train, (m) TCGA-Test, (n) E-MTAB-1980, and (o) E-MTAB-3267 cohorts.

The TCGA cohort was randomly divided into training and test groups according to the sample size. We found that in all data sets, the survival rate of KIRC patients in the low-risk group was higher than that in the high-risk group (TCGA, median survival 1137 days (low-risk) versus 970 days (high-risk); TCGA-Train, median survival 1203 days (low-risk) versus 1150 days (high-risk); TCGA-Test, median survival 1120 days (low-risk) versus 911 days (high-risk); E-MTAB-1980, median survival 1530 days (low-risk) versus 1455 days (high-risk); E-MTAB-3267, median survival 365 days (low-risk) versus 349 days (high-risk)). To test the predictive power and accuracy of the constructed risk model, ROC curve analysis was used to measure the 1-, 3-, and 5-year survival rates of each dataset. The corresponding 1-, 3-, and 5-year area under curves (AUCs) in the TCGA cohort were 0.656, 0.668, and 0.676, respectively; in the TCGA-Train cohort, they were 0.753, 0.728, and 0.705, respectively; in the TCGA-Test cohort, they were 0.580, 0.615, and 0.643, respectively; in the E-MTAB-1980 cohort, they were 0.745, 0.675, and 0.719, respectively; and in the E-MTAB-3267 cohort, they were 0.562, 0.620, and 0.585, respectively (Figure 3).

### 3.4. Correlation between the Risk Model and Clinical Characteristics

**Figure 4:**
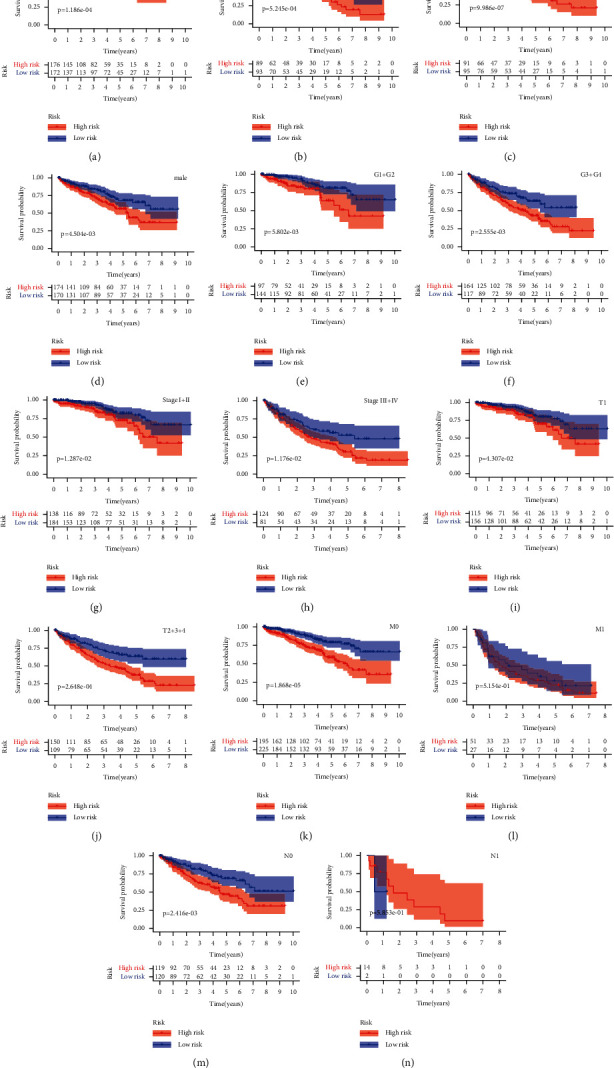
Correlation between the risk model and clinical characteristics. Stratification analysis for the risk model in (a) age ≤65, (b) age >65, (c) female, (d) male, (e) G1 + G2, (f) G3 + G4, (g) stage I + II, (h) stage III + IV, (i) T1, (j) T2+3 + 4, (k) M0, (l) M1, (m) N0, and (n) N1 in TCGA.

To test the relationship between the risk model and clinical characteristics, we used Fisher's test to measure the differences between the distribution of risk scores and common clinical characteristics. The results of this analysis are shown in Tables 2 and 3. We found that the high- and low-risk groups showed significant differences in grade, disease stage, tumor size (T), metastases (M), and lymph node involvement (N). The risk model was also highly predictive of these common clinical features. Fisher's test on the age and sex showed no statistical significance, but the survival rates of patients in the low-risk group were significantly higher than those in the high-risk group in men and women aged ≤65 and > 65 years. In the early stage I + II, late stage III + IV, grade I + II, and grade III + IV, as well as T1, T2 + 3 + 4, M0, and N0 of the TMN system, the survival rates of the patients in the low-risk group were also significantly higher than those in the high-risk groups. However, these findings may have been limited by the sample size. Furthermore, M1 and N1 did not show significant differences between the high- and low-risk groups (Figure 4).

Next, we conducted single-factor and multifactor Cox analyses on the risk score and clinical characteristics and discovered the potential of the score as an independent prognostic factor for KIRC patients (Supplementary Figure 2).

### 3.5. Omics Characteristics of the High- and Low-Risk Groups

**Figure 5:**
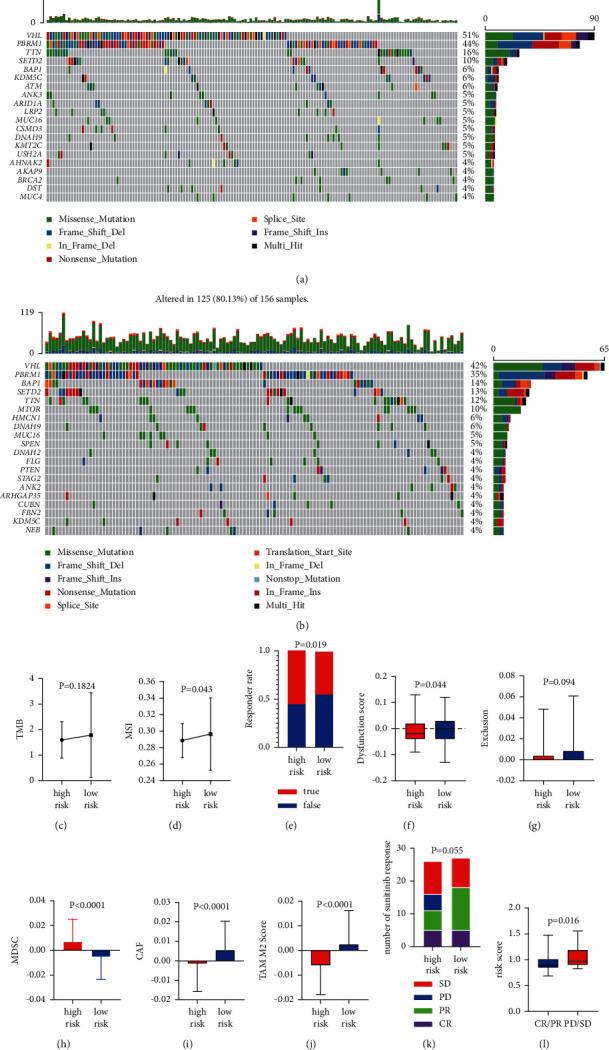
Somatic mutations in the low- and high-risk groups and the potential for immunotherapy. Landscape of somatic mutations in the (a) low- and (b) high-risk groups of the top 20 genes. (c) Tumor mutation burden (TMB) and (d) microsatellite instability (MSI) in the low- and high-risk groups. (e) Predictable responder rate, (f) dysfunction score, (g) exclusion score, (h) myeloid-derived suppressor cells (MDSC), (i) tumor-associated fibroblasts (CAF), and (j) tumor-associated macrophages (TAM) M2 score of the low- and high-risk groups in the TCGA cohort. (k) Therapy response of sunitinib in the E-MTAB-3267 cohort and distribution of risk score with different response populations. (l) Risk score between patients with CR/PR and PD/SD; CR, complete response; PR, partial response; PD, progressive disease; SD, stable disease.

Several studies have shown that immune checkpoint inhibitor (ICI) treatment benefits some patients with metastatic cancer, and findings in certain cancer types indicate that tumor mutation burden (TMB) may predict the clinical response to ICI [13–15]. To explore the omics characteristics of the high- and low-risk groups and look for potential treatment targets, we constructed a panoramic view of the mutations in both groups and performed an immune examination using the TIDE algorithm [16] for ICI treatment prediction. We found that in the top 20 most frequently mutated genes, the mutation rates of the tumor suppressor gene VHL in the high- and low-risk groups were 51% and 42%, respectively, of PBRM1, 44% and 35%, respectively, of TTN, 16% and 12%, respectively, and of SETD2, 10% and 13%, respectively. In addition, BAP1 (14%) and MTOR (10%) had higher mutation frequencies in the high-risk group (Figures 5(a) and 5(b)).

The total number of somatic mutations identified was normalized to the corresponding exon coverage of the KIRC-VarScan panel in megabases. The low-risk group had higher TMB and MSI scores than the high-risk group. However, in terms of actual TIDE prediction results, the high-risk group had a higher response rate to immune checkpoint inhibitors, but lower dysfunction and exclusion scores than the low-risk group, reflecting the tumor escape mechanisms. In terms of the three cell types that restrict T cell infiltration in tumors, the high-risk group had higher scores for myeloid-derived suppressor cells (MDSCs), tumor-associated fibroblasts (CAF), and tumor-associated macrophages (TAM) than the low-risk group. Furthermore, in the sunitinib drug trial cohort of E-MTAB-3267, the disease progression of patients in the low-risk group was significantly lower than that of patients in the high-risk group (Figures 5(k) and 5(l)).

### 3.6. Correlation between Genes and Immune Cells in the Risk Model

**Figure 6:**
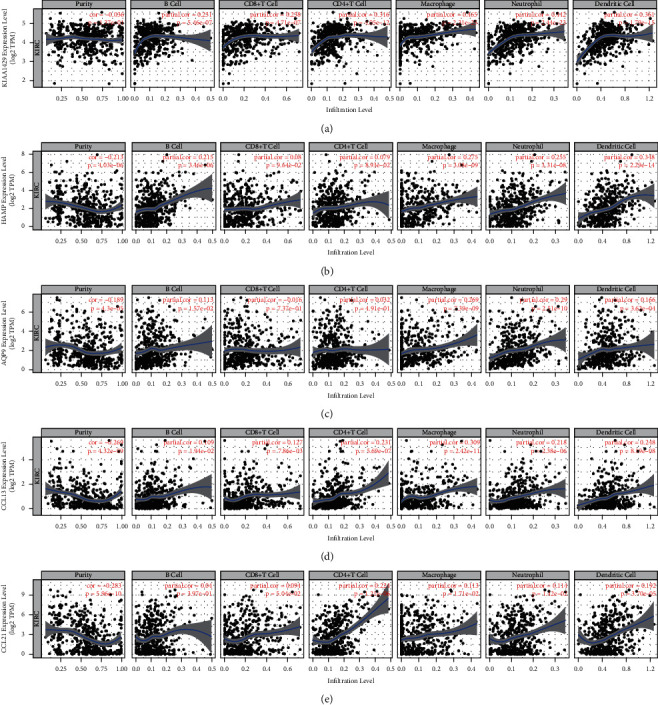
Correlation between genes and immune cells in the risk model. Correlation of (a) KIAA1429, (b) HAMP, (c) AQP9, (d) CCL13, and (e) CCL21 with tumor purity and the levels of B cells, CD8 T cells, CD4 T cells, macrophages, neutrophils, and dendritic cells.

To further explore the regulatory relationship between the previously described immune genes and immune cells in the risk model, we used TIMER to verify the correlation between the five immune genes and the purity of the main immune cells and tumors. The results showed that KIAA1429, HAMP, AQP9, CCL13, and CCL21 were significantly negatively correlated with tumor purity, and KIAA1429 was highly correlated with B cells, CD8 T cells, CD4 T cells, macrophages, neutrophils, and dendritic cells. HAMP was also highly correlated with B cells, macrophages, neutrophils, and dendritic cells, while AQP9 was highly correlated with macrophages and neutrophils. Lastly, CCL21 was highly correlated with CD4 T cells and neutrophils (Figure 6).

### 3.7. Kaplan–Meier Survival Validation of Genes in the Risk Model

Multivariate Cox regression analysis revealed that the five immune genes in the risk model are independent prognostic factors for ccRCC. We conducted Kaplan–Meier survival analysis for each gene and found that AQP9, KIAA1429, and HAMP had a significant impact not only on the overall survival of ccRCC patients, but also on progression-free survival. Among them, the high expression of AQP9 and HAMP indicates a poor prognosis for ccRCC.=, whereas KIAA1429 is a protective factor for ccRCC, and its high expression indicates a good prognosis of ccRCC. Moreover, we also found that these five immune genes have different degrees of methylation in the cancer tissues of ccRCC patients compared to that in the corresponding normal tissues. AQP9 and KIAA1429 were upregulated, and CCL13, CCL21 and HAMP were downregulated (Supplementary Figure 3).

## 4. Discussion

With the rapid development of high-throughput sequencing, it is easier for researchers to explore and identify new therapeutic targets for disease or cancer management. However, most current research focuses on analyzing the differential markers between cancer cells and adjacent or normal tissues. This line of thinking does not take into account the differences between the various classifications of cancer cells and tumor microenvironments. In this study, we classified patients with ccRCC into either high- or low-immunity groups and identified 132 differential immune genes between them. Finally, through multivariate Cox regression analysis, we identified five genes, AQP9, KIAA1429, HAMP, CCL13, and CCL21, for use in a risk prognosis model, which was found to be highly predictive of clinical features and response to immunotherapy.

Aquaporin-9, the protein encoded by the AQP9 gene, has been found to be carcinogenic in many tumors [17]. It is a membrane channel protein that allows the penetration of small solutes, including glycerol, urea, and nucleobases. However, its prognostic value in patients with ccRCC still needs to be clarified. In glioblastomas, the expression of AQP9 mRNA is mainly caused by the infiltration of AQP9-expressing leukocytes into tumor sites. Jelen et al. [18] and Vogl et al. [19] confirmed that AQP9 mRNA is coregulated with transcripts encoding natural immune response components, such as complement components and molecules that mediate response to bacterial lipopolysaccharide. The expression of AQP9 also appears to be highly correlated with the expression of calcin A and B (MRP8/S100A8 and MRP14/S100A9, respectively). These mRNAs encode proteins that form calmodulin A-calmodulin B dimers, which act as ligands for the toll-like receptor 4. In our study, AQP9 was mainly associated with macrophage, centrocyte-related cytokine, and chemokine activity in ccRCC. Therefore, we speculate that the mechanism of AQP9 expression in ccRCC is similar to that in acute leukemia.

HAMP is a gene that encodes hepcidin, a protein that is involved in the maintenance of iron homeostasis, which is necessary for regulating iron storage in macrophages and iron absorption in the intestines [20]. Wang et al. [21] reported that the regulation of iron metabolism plays an important role in promoting cell proliferation in ovarian cancer. Changes in many functional genes affect the process of iron metabolism, such as an increase in the expression of TFR1, DMT1, and HAMP (Supplementary Figure 1) and a decrease in the expression of FPN. This leads to high intracellular iron concentrations and high FTL content, promoting the development of advanced tumors. The iron chelator deferoxamine is used to inhibit tumors by depleting the intracellular iron pool in tumor cells and preventing stem cell growth. Although the specific regulatory mechanism of HAMP in ccRCC is unknown, the abovementioned therapies provide useful insight into the treatment of ccRCC in the future.

KIAA1429 is a gene that encodes the M6A methyltransferase-related protein, which is also known as the vir-like m6 methyltransferase-related protein (VIRMA). This protein is known to be important for the establishment of the cell m6A spectrum. This gene has also been reported to be associated with the development of various cancers [22–25]. It is known to have different regulatory mechanisms in different cancers. At the cellular level, VIRMA inhibits the cell viability and proliferation of PC-3 cells and inhibits malignant phenotypes by reducing its migration and invasion activities [22]. In hepatocellular carcinoma, GATA3 has been identified as a direct downstream target of the KIAA1429-mediated m6A modification. In this process, KIAA1429 induces m6A methylation on the 3'UTR of the GATA3 pre-mRNA, leading to the separation of the RNA-binding protein HuR and the degradation of the GATA3 pre-mRNA, inducing tumor growth and metastasis [23]. In breast cancer, KIAA1429 also regulates downstream target genes such as CDK1, which encodes the protein cyclin-dependent kinase 1, a protein that exhibits a carcinogenic effect [25]. While its overexpression in cancer tissues is typically associated with poor prognosis in patients with cancer, we have found that its overexpression is associated with a good prognosis in ccRCC patients. This shows the complexity of its role in various types of cancers.

Chemokines are a homologous group of proteins with different functions. They directly mediate the migration and activation of leukocytes and play a role in angiogenesis regulation. They are also involved in the maintenance of immune homeostasis and structures of the secondary lymphoid organs. CCL13, also known as the monocyte chemotactic protein, is a chemokine that induces various activities in its target cells. This protein plays an important role in the innate immune response. When epithelial cells are activated by cytokines or pathogen-associated molecular patterns through toll-like receptors, CCL13 is released together with other chemokines through NF*κ*B activation [26]. The secretion of these chemokines leads to inflammation-related events, such as the overexpression of endothelial cell adhesion molecules [27]. For example, in fibroblasts and smooth muscles, CCL13 induces cell proliferation related to remodeling [28] and dendritic cell activation [29]. Therefore, CCL13 can be used as a key molecule to allow selective recruitment and activation of certain cell lineages to inflammatory tissues. This suggests that blocking the effects of CCL13 may be used as a new strategy for the development of drugs with anti-inflammatory activity.

CCL21 has been identified as a lymphatic chemokine that is expressed constitutively by high endothelial venules, lymph nodes, lymph vessels, and interstitial cells of the spleen and appendix [30]. This protein binds to the chemokine receptor CCR7 and acts as a chemoattractant for mature DCs, naive T cells, and memory T cells. Through the activation of the G protein-coupled CCR7 transmembrane receptor, CCL21 mediates the recruitment of the abovementioned cells to the T cell area of secondary lymphoid organs, promoting T cell activation. CCL21 also costimulates the expansion of CD4+ and CD8+ T cells and induces Th1 polarization. The immunosuppressive cell population, consisting of CD4+, CD25+, and regulatory T cells, is less responsive to CCL21-induced migration and unresponsive to CCL21 costimulation [31]. These CCL21 functions attract as well as costimulate the proliferation, differentiation, and activation of naive T cells. This indicates that CCL21 is a key molecule that triggers T cell responses and may have therapeutic significance in ccRCC treatment.

In this study, we focused on exploring the heterogeneity of the tumor microenvironments from patients with ccRCC, identified two immune subtypes of ccRCC, and identified five immune genes (AQP9, KIAA1429, HAMP, CCL13, and CCL21) from the high and low subgroups that were used to construct a risk model.

Furthermore, a new immune subtype of ccRCC was identified based on the TCGA cohort, which was also verified in other cohorts. A risk prognosis model consisting of five immune genes, AQP9, KIAA1429, HAMP, CCL13, and CCL21, was also constructed based on the identified subtypes. The activities of cytokines and chemokines, including the recruitment of immune cells, such as B cells, dendritic cells, macrophages, and neutrophils, the presentation of antigens, and the activation of T cells, may provide possible targets for the development of new immunotherapy methods in the future.

We recognize several limitations in our study. First, in the process of screening immune genes for the risk model construction, other important immune genes may have been missed. Second, the sample size of our risk model was still limited. Based on our in-depth analysis of ccRCC microenvironment transcriptome data, further in vivo and in vitro experiments are needed to verify the biological functions and mechanisms of the genes in our developed signature of ccRCC immune heterogeneity.

## 5. Conclusions

In summary, by using TCGA data to evaluate the immune genes of 530 patients with ccRCC, we developed an effective risk score based on five immune genes that has clinical potential in the prognosis of ccRCC. In addition, these five immune genes reveal the huge inherent heterogeneity of the ccRCC tumor microenvironment, as well as the potential and possibility of treatment with immune checkpoint inhibitors. Furthermore, in the clinical trial of the E-MTAB-3267 cohort involving sunitinib treatment of metastatic ccRCC, our risk score also shows the ability to predict whether the disease will progress or not, which will promote the practical application of precision medicine, as well as provide new insights for the current treatment of ccRCC.

## Figures and Tables

**Table 1:** Coefficients of the five immune genes used to construct the risk signature, as identified from the multivariate Cox regression analysis in the TCGA cohort.

Gene	Coef	HR	HR.95L	HR.95H	*P* value
CCL13	0.118931	1.126292	1.043501	1.215651	0.002265
KIAA1429	−0.177806	0.837105	0.734482	0.954066	0.007707
AQP9	0.015864	1.015991	1.002345	1.029822	0.021474
CCL21	0.006363	1.006384	1.002058	1.010728	0.003784
HAMP	0.261579	1.298979	1.155972	1.459678	1.10*E* − 05

Coef: coefficient; HR: hazard ratio.

**Table 2:** Clinical features of the low- and high-risk groups in the TCGA cohort.

Clinical parameters		Total	High risk	Low risk	*P* value
		530	265	265	
Sex	Female	186	91	95	0.7848238
	Male	344	174	170	

Age	≤65	348	176	172	0.7837531
	>65	182	89	93	

Grade	G1 + G2	241	97	144	0.1655402
	G3 + G4	281	164	117	
	Gx or unknown	8	4	4	

Stage	Stage I + II	322	138	184	9.18*E *− 05
	Stage III + IV	205	124	81	
	Unknown	3	3	0	

T	T1	271	115	156	0.0005092
	T2 + 3 + 4	259	150	109	

M	M0	420	195	225	0.0048623
	M1	78	51	27	
	Mx or unknown	32	19	13	

N	N0	239	119	120	0.0088965
	N1	16	14	2	
	Nx	275	132	143	

T, tumor; M, metastasis; N, lymph nodes. *P* values were obtained by Fisher's test.

**Table 3:** Clinical features of the low- and high-risk groups in the E-MTAB-1980 cohort.

Clinical parameters		Total	High risk	Low risk	*P* value
		101	50	61	
Sex	Female	24	12	12	1
	Male	77	38	39	

Age	≤65	57	35	22	0.01168567
	>65	44	15	29	

pT	pT1	68	32	36	0.6215551
	pT2-4	33	18	15	

N	N0	94	45	49	0.4175018
	N1-2	7	5	2	

M	M0	89	43	46	0.7307897
	M1	12	7	5	

Fuhrman grade	1 + 2	72	35	37	0.9594934
	3 + 4	27	14	13	
	Undetermined	2	1	1	

pT, pathological tumor; M, metastasis; N, lymph nodes. *P* values were obtained by Fisher's test.

## Data Availability

The datasets generated or analyzed in this study can be found in the TCGA (http://portal.gdc.cancer.gov/repositary) and ArrayExpress (http://www.ebi.ac.uk/arrayexpress/) databases.

## Supplementary Materials

Supplementary Materials Supplementary Figure 1. Functional enrichment analysis of 67 immune genes. Gene ontology (GO) terms: molecular function (MF), biological process (BP), and cellular component (CC); (B) Kyoto Encyclopedia of Genes and Genomes (KEGG) analysis of 67 immune genes. Supplementary Figure 2. Independent prognostic analysis of risk score and clinical characteristics. (a) Univariate and (b) multivariate Cox regression analyses of risk score and clinical characteristics in the TCGA cohort. (c) Univariate and (d) multivariate Cox regression analyses of risk score and clinical characteristics in the E-MTAB-1980 cohort. T, tumor; M, metastasis; N, lymph nodes. Supplementary Figure 3. Kaplan–Meier survival validation of the five genes in the risk model. (a–e) Overall survival when AQP9, CCL13, CCL21, HAMP, or KIAA1429 is expressed in ccRCC in the TCGA cohort; (f–j) progression-free survival when AQP9, CCL13, CCL21, HAMP, or KIAA1429 is expressed in ccRCC in the TCGA cohort; (k–o) methylation differences in AQP9, CCL13, CCL21, HAMP, and KIAA1429 between ccRCC and normal tissues in the TCGA cohort. .
