# Supplementary material for: Development of an Individualized Immune Prognostic Signature for Clear Cell Renal Cell Carcinoma through the Identification of Differential Immune Genes
Source: J Oncol. 2021 Aug 11;2021:9587084. doi: 10.1155/2021/9587084 (PMC8376451; doi:10.1155/2021/9587084)
Supplement: Supplementary Materials — Supplementary Figure 1. Functional enrichment analysis of 67 immune genes. Gene ontology (GO) terms: molecular function (MF), biological process (BP), and cellular component (CC); (B) Kyoto Encyclopedia of Genes and Genomes (KEGG) analysis of 67 immune genes. Supplementary Figure 2. Independent prognostic analysis of risk score and clinical characteristics. (a) Univariate and (b) multivariate Cox regression analyses of risk score and clinical characteristics in the TCGA cohort. (c) Univariate and (d) multivariate Cox regression analyses of risk score and clinical characteristics in the E-MTAB-1980 cohort. T, tumor; M, metastasis; N, lymph nodes. Supplementary Figure 3. Kaplan–Meier survival validation of the five genes in the risk model. (a–e) Overall survival when AQP9, CCL13, CCL21, HAMP, or KIAA1429 is expressed in ccRCC in the TCGA cohort; (f–j) progression-free survival when AQP9, CCL13, CCL21, HAMP, or KIAA1429 is expressed in ccRCC in the TCGA cohort; (k–o) methylation differences in AQP9, CCL13, CCL21, HAMP, and KIAA1429 between ccRCC and normal tissues in the TCGA cohort. . [file 9587084.f1.pdf]

Supplementary Material

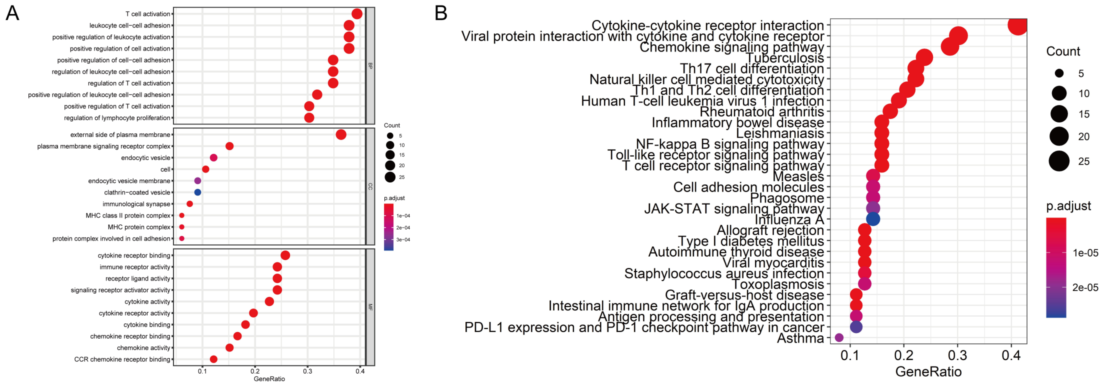

Supplementary Figure 1. Functional enrichment analysis of 67 immune genes.

(A) Gene Ontology (GO) term in molecular function (MF), biological process (BP) and cellular component (CC); (B) Kyoto Encyclopedia of Genes and Genomes (KEGG) for 67 immune genes.

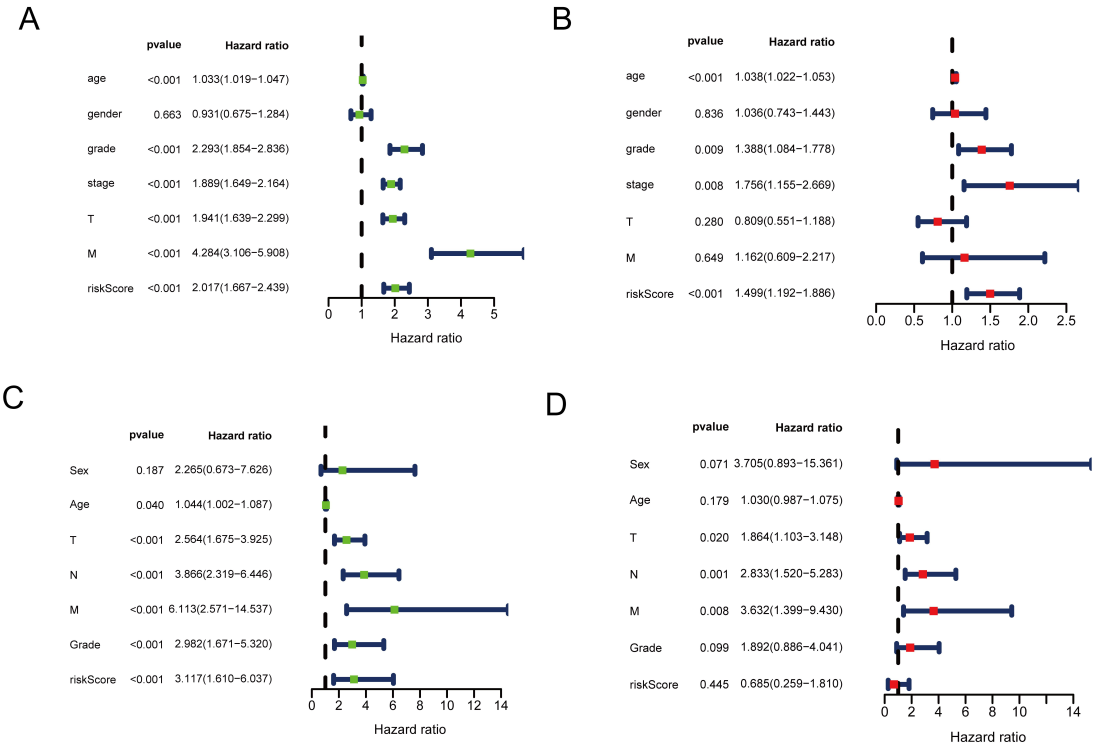

Supplementary Figure 2. Independent prognostic analysis of risk score and clinical characteristics. (A) Univariate and (B) multivariate Cox regression analysis of risk score and clinical characteristics in TCGA cohort, (C) Univariate and (D) multivariate Cox regression analysis of risk score and

clinical characteristics in E-MTAB-1980 cohort. T, tumor; M, metastasis; N, lymph nodes.

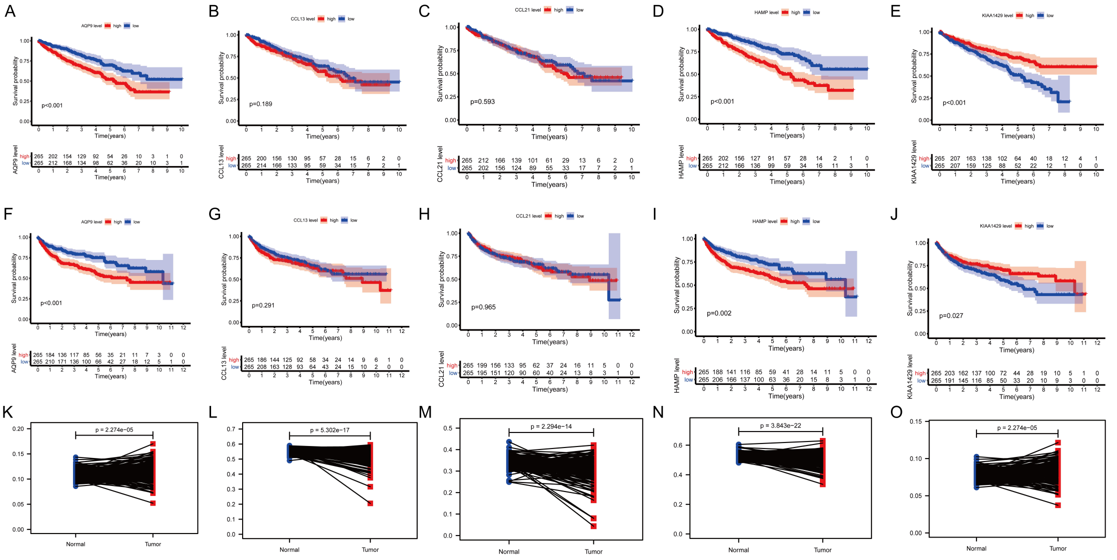

Supplementary Figure 3. Kaplan-Meier survival validation of five genes in the risk model. (A-E) Overall survival for *AQP9*, *CCL13*, *CCL21*, *HAMP* and *KIAA1429* in ccRCC of TCGA; (F-J) progression-free survival for *AQP9*, *CCL13*, *CCL21*, *HAMP* and *KIAA1429* in ccRCC of TCGA; (K-O) methylation differences between tumor and normal tissues of ccRCC for *AQP9*, *CCL13*, *CCL21*, *HAMP* and *KIAA1429* in TCGA.
